# Strategies to Increase Latino Immigrant Youth Engagement in Health Promotion Using Social Media: Mixed-Methods Study

**DOI:** 10.2196/publichealth.9332

**Published:** 2018-12-19

**Authors:** Elizabeth Louise Andrade, W Douglas Evans, Nicole Barrett, Mark Cameron Edberg, Sean D Cleary

**Affiliations:** 1 Department of Prevention and Community Health Milken Institute School of Public Health The George Washington University Washington, DC United States; 2 Department of Epidemiology and Biostatistics Milken Institute School of Public Health The George Washington University Washington, DC United States

**Keywords:** social media, health promotion, Latinos, immigrants, adolescent, Hispanic Americans, emigrants and immigrants, adolescent health

## Abstract

**Background:**

Generating participant engagement in social media applications for health promotion and disease prevention efforts is vital for their effectiveness and increases the likelihood of effecting sustainable behavior change. However, there is limited evidence regarding effective strategies for engaging Latino immigrant youth using social media. As part of the Avance Center for the Advancement of Immigrant/Refugee Health in Washington, DC, USA, we implemented Adelante, a branded primary prevention program, to address risk factors for co-occurring substance use, sexual risk, and interpersonal violence among Latino immigrant adolescents aged 12 to 19 years in a Washington, DC suburb.

**Objective:**

The objectives of this study were to (1) characterize Adelante participant Facebook reach and engagement and (2) identify post content and features that resulted in greater user engagement.

**Methods:**

We established the Adelante Facebook fan page in October of 2013, and the Adelante social marketing campaign used this platform for campaign activities from September 2015 to September 2016. We used Facebook Insights metrics to examine reach and post engagement of Adelante Facebook page fans (n=743). Data consisted of Facebook fan page posts between October 1, 2013 and September 30, 2016 (n=871). We developed a 2-phased mixed-methods analytical plan and coding scheme, and explored the association between post content categories and features and a composite measure of post engagement using 1-way analysis of variance tests. *P*<.05 determined statistical significance.

**Results:**

Posts on the Adelante Facebook page had a total of 34,318 clicks, 473 comments, 9080 likes or reactions, and 617 shares. Post content categories that were statistically significantly associated with post engagement were Adelante program updates (*P*<.001); youth achievement showcases (*P*=.001); news links (*P*<.001); social marketing campaign posts (*P*<.001); and prevention topics, including substance abuse (*P*<.001), safe sex (*P*=.02), sexually transmitted disease prevention (*P*<.001), and violence or fighting (*P*=.047). Post features that were significantly associated with post engagement comprised the inclusion of photos (*P*<.001); Spanish (*P*<.001) or bilingual (*P*=.001) posts; and portrayal of youth of both sexes (*P*<.001) portrayed in groups (*P*<.001) that were facilitated by adults (*P*<.001).

**Conclusions:**

Social media outreach is a promising strategy that youth programs can use to complement in-person programming for augmented engagement. The Latino immigrant youth audience in this study had a tendency toward more passive social media consumption, having implications for outreach strategies and engagement measurement in future studies. While study findings confirmed the utility of social marketing campaigns for increasing user engagement, findings also highlighted a high level of engagement among youth with posts that covered casual, day-to-day program activity participation. This finding identifies an underexplored area that should be considered for health messaging, and also supports interventions that use peer-to-peer and user-generated health promotion approaches.

## Introduction

### Background

Social media has numerous applications for health promotion and disease prevention efforts, and the benefits of using social media for public health communication have been documented [1-4]. Results of health promotion efforts delivered via social media or using digital technologies have demonstrated increases in health knowledge [5-7], have assisted users with chronic disease management [8], and have led to improvements in health-related practices [9,10]. The use of digital technologies, such as mobile phone-based interventions, has also achieved outcomes such as increased adherence to treatments and increased engagement with behavior change interventions [11]. Social media platforms have been highly useful for increasing peer, social, and emotional support [12-22] and for reaching marginalized and underserved audiences while also connecting low-literacy groups [23-32] to information and resources.

Generating participant engagement is vital for effective health promotion whereby, through captivating an audience’s attention, we can go beyond simply reaching an audience to influencing sustainable changes in health behaviors [33]. However, engagement is an understudied area, and there are gaps in the literature regarding strategies that lead to greater engagement, especially among underserved populations [34-37]. Research has indicated that participants are more likely to be engaged when there is connectivity and multiple points of contact between participant and program; participation is easy yet rewarding; and participants are interested in and identify with the messages being conveyed or a program’s brand [34]. Engagement of youth populations plays an integral role in healthy cognitive, social, and emotional development; can provide opportunities for the acquisition of skills and increased confidence; and can result in youth contributing to their communities [35-45].

Social media has many attributes that may potentially increase engagement for health promotion and disease prevention programs. First and foremost, social media is ubiquitous and can reach very large audiences, enabling broad dissemination of health information and messaging [46-49]. Social media messages can be highly personalized and tailored to the interests of specific audiences, including peer groups. Social networks can also be leveraged to further amplify dissemination, increase interaction with other users from a targeted group [47,50-53], and augment the credibility of information shared between known contacts [54,55]. Importantly, social media can be used to augment in-person programming to increase the effectiveness of health promotion programs seeking to change health-related behaviors [2,4,56-59].

Similar to the general public, youth populations appear to be amenable to receiving health-related information on social media [31,60-62]. Thus, understanding tactics that can increase engagement in health promotion programs for young audiences via social media will ensure effective use of this tool. Identifying predictors of social media engagement can guide the development of content and use of features that have high appeal for young audiences. Past research has identified some strategies for successful user engagement, including encouraging high levels of social media activity, individual user interaction, and interaction through posing questions or calls to action; using multimedia content; highlighting celebrity involvement; tagging users in posts; targeting messages to specific audiences; using strategic message framing; leveraging targeted outreach campaigns; using humor or shock appeals; and storytelling. However, few studies have focused on social media engagement for Latino immigrant adolescents [63-68].

Latino adolescents are an important and growing population in the United States. The United States Census Bureau estimated that, by 2050, Latinos will constitute 34% of the US adolescent population between the ages of 10 and 19 years [69]. Yet Latino adolescents continue to experience numerous health disparities, have more limited engagement in health promotion, and have lower rates of health care utilization [70-80]. Despite pervasive access to mobile technology and widespread use of social media by Latino adolescents, there is little evidence establishing the best ways to engage with this audience using social media, highlighting the importance of further exploration of this area [1,81-90]. The literature base exploring engagement through social media of Latino *immigrants*, a subgroup of Latinos who have more recently immigrated to the United States, is even more limited. The immigrant subgroup should be distinguished from Latino subgroups that have a more established presence in the United States, sometimes for generations. Recent immigrants tend to be harder to reach with health promotion programs, since they are more likely to experience a unique set of risk factors that contribute to health disparities [91-93]. Social media outreach has the potential to engage Latino immigrant adolescents in health promotion efforts, contributing to youth-centered initiatives that use tested engagement strategies and more meaningful experiences for this group of young people [38].

### Objective

In this study, we explored Facebook engagement for a branded primary prevention intervention for Latino immigrant youth, called Adelante [94]. The study aims were to (1) characterize Adelante participant Facebook reach and engagement and (2) identify post features and content that resulted in greater user engagement. Ultimately, we sought to formulate predictors of user engagement that would inform future prevention programming and youth outreach strategies using social media.

## Methods

### Adelante Youth Intervention and Social Media

We developed and implemented the 4-year Adelante primary prevention program to address risk factors for co-occurring substance use, sexual risk, and interpersonal violence among Latino immigrant adolescents, aged 12 to 19 years, living in Langley Park, Maryland, a community close to Washington, DC [94]. Adelante was grounded in an adapted positive youth development (PYD) framework, using a multilevel, asset-based approach for risk prevention [95,96]. Adelante addressed the following PYD constructs: *competence*, *confidence*, *connection*, and *contribution* (detailed elsewhere) [94,97]. The Adelante intervention consisted of in-person youth and parent programming, case management for high-risk youth and their families, and a social marketing campaign [98]. Adelante also applied innovative engagement strategies, whereby in-person activities intersected with the Adelante social media network, and participants also created user-generated digital media content informed by the Adelante brand and a foundation laid by previous activities and research [94,97-103].

We applied social marketing and branding principles for the development and implementation of Adelante components that sought to engage participants in the Adelante brand and program, increase receptivity to prevention messages, and, ultimately, improve Latino adolescent risk-preventive attitudes, norms, and behaviors [104-106]. As part of the overall engagement strategy, we implemented a 1-year social marketing campaign [98] that incorporated digital and print media advertisements, social media outreach, and the creation of user-generated content in collaboration with Adelante youth ambassadors [102]. Given youths’ affinity for digital media, the Adelante program had an active social media presence as a strategy for disseminating prevention messages, engaging the target youth audience in the Adelante brand, and increasing peer-to-peer and peer-to-program connectivity.

This study built on previous research by using Facebook Analytics tools (Facebook, Inc, Menlo Park, CA, USA) to identify post content and features that were associated with higher engagement among fans of the Adelante program’s Facebook page. This study expanded our understanding of predictors of engagement that can be applied to future health promotion and disease prevention initiatives with similar Latino immigrant audiences.

### Study Population

The study population comprised fans of the Adelante Facebook page, which predominantly included Latino immigrant adolescents aged 12 to 19 years. We recruited fans from the in-person Adelante program and their immediate social networks, which we implemented in the community of Langley Park, Maryland. Langley Park is a low-income, mostly foreign-born (67.6%) Latino (80%) community [107]. A recent study by Cleary and colleagues estimated that, among adolescent Latinos aged 12 to 17 years, 66% were recently arrived immigrants, having lived in the United States for 3 years or less, with a large representation from El Salvador (46.53%), Guatemala (32.86%), and Honduras (10.41%; SC, unpublished data, 2017).

### Adelante Program Facebook Fan Page

The Adelante Facebook fan page was established in October 2013, and the Adelante social marketing campaign used this platform for a portion of the campaign’s activities from September 2015 to September 2016. We used the fan page for ongoing program-related posts, such as recruiting for programs or events; showcasing programmatic activities; disseminating information about social issues or initiatives; sharing information about health, social services, or educational resources and opportunities; and providing opportunities for Adelante staff or peers to provide social support to participants. The social marketing campaign disseminated health-risk and prevention information via social media (related to substance use, sexual risk, and interpersonal violence), and sought to further engage youth through targeted outreach and messaging using advertisements and user-generated video content that featured Adelante youth; contests; highlights of youth stories and achievements; and links to news stories of interest, websites, blog posts, and other resources. We also occasionally boosted posts to explore the utility of this strategy for increasing reach and engagement.

### Data Collection and Metrics

Data for this study consisted of Facebook posts on the Adelante fan page between October 1, 2013 and September 30, 2016. We used Facebook Insights metrics to examine the reach and post engagement of Adelante Facebook page fans. Metrics for reach were number of page fans, number of posts, total reach, organic reach, and paid reach. Facebook defines reach as “the number of unique people who see a post” and engagement as “reacting to, sharing, or commenting or clicking on any content” [108]. Paid reach refers to the number of unique people who see a post as a result of advertisements, whereas organic reach does not involve advertisements. We created a composite post engagement dependent variable by summing post clicks, reactions, comments, and shares. Adelante research methods and protocols underwent the George Washington University Institutional Review Board review and approval process for the protection of human subjects (study number 111139; Washington, DC, USA).

### Data Analysis

We developed a 2-phased mixed-methods analysis plan that enabled us to combine quantitative Facebook analytics and assessment of post features with qualitative data from Facebook posts (text, media, and graphics). We exported Facebook data into an Excel database version 2013 (Microsoft Corporation) using NCapture (QSR International Pty Ltd). In phase 1, we coded the data quantitatively using a coding scheme that assigned numeric values to post features, such as purpose of the post or language used (Spanish, English, or both; Table 1). In phase 2, we qualitatively coded post text and media, as well as text and media of external links included in the post.

**Table 1:** Post content and features coding scheme (phases 1 and 2).

Phase and content		Features	
**Phase 1**			
	Language used		English, Spanish, bilingual
	Post tone		Positive, negative
	Purpose of post		Program announcement, program activity sharing, health or social service promotion, internship or educational opportunity advertisements, health education or promotion, contest, youth achievement or story highlight, news story sharing, awareness raising or social issue advocating, and campaign messaging
**Phase 2**			
	Prevention topic		Sexual health: sexually transmitted diseases, pregnancy and birth control, safe sex (condom use, abstinence), risk prevention; violence: partner violence, bullying, peer violence or fighting, risk prevention; substance abuse: factual information, risks of substance use, risk prevention; mental health: factual information, symptoms, sources of help, risk prevention
	Positive youth development framework construct		Confidence; contribution (attitudes and action); competence (athletics, civic action, school, family, multicultural efficacy); connection (romantic partner, community, social and cultural, school, friends and peers, family)
	People portrayed		Individuals or groups; gender (male, female, both male and female); adults and youth

Posts were coded according to prevention topic, PYD construct, people portrayed in the post, and whether the post was boosted or not (Table 1). For the qualitative coding, we used NVivo software version 16 (QSR International Pty Ltd). After qualitative coding was complete, we used NVivo software to convert qualitative coding to a quantitative format. We then combined this quantitative dataset with the quantitative dataset from phase 1 for analysis.

Following data coding, we examined the distribution for the post engagement dependent variable and all independent variables. All variables were not normally distributed but did have a similar pattern (or shape) of distribution. Given the nonnormal distribution, and with all of the assumptions being met, we decided to use the nonparametric Mann-Whitney *U* test to identify statistically significantly different median scores between groups (yes or no for a particular post content or feature) for post content category or feature (dichotomous independent variables) by post engagement (continuous dependent variable). We conducted the analysis using IBM SPSS version 19 (IBM Corporation). *P*<.05 determined statistical significance.

## Results

The Adelante Facebook page reached a total of 743 fans with 871 posts. The total reach was 247,212 users, including an organic reach of 163,698 unique users (representing 850 posts) and a paid reach of 83,514 unique users (representing 21 posts). A total of 213 posts were made as a part of the 1-year social marketing campaign. Regarding overall engagement metrics, there were 34,318 post clicks, 473 post comments, 9080 post likes or reactions, and 617 post shares.

As Table 2 shows, posts that provided updates about program activities that had occurred recently garnered a lot of interest from Adelante Facebook fans. These posts tended to occur during, or immediately following, program activities, and the posts usually contained photos of youth participating in group activities that were facilitated by an adult. The images in program update posts also portrayed youth enjoying time with their peers in groups of male and female youth, which reflects scenarios of how youth in the study community most commonly socialize. Statistically significant differences in engagement for posts targeting PYD constructs of connection-peer and competence-physical activity can be explained by youths’ interest in a highly popular program activity—soccer teams and tournaments—which were promoted on social media (Table 3). Fans were also interested in seeing youth participating in career workshops and internships (competence-workplace) and youth contributing to their communities through volunteering or community cleanups (contribution-action). Posts that showcased Adelante youths’ personal stories and achievements were also engaging to Facebook fans.

Posts that were part of the Adelante social marketing campaign were also engaging to youth. These posts often contained photos, videos, and branded advertisements that portrayed local Adelante youth, whom participants knew firsthand, thus likely increasing youth interest. These posts also included content that promoted PYD-informed messages, and disseminated information related to health promotion and risk prevention. The most engaging topics included in posts were substance abuse prevention (a main focus of the Adelante program), safe sex and sexually transmitted disease prevention, and violence prevention—specifically, fighting. Topics that were less engaging were mental health, pregnancy prevention, bullying, and partner violence, which are very surprising given our experience working with this population.

**Table 2:** Results: post content variables and user engagement.

Post content (independent variable)		Posts, n (%)	*P* value (dependent variable)
**Post purpose**			
	Program announcement or reminder	229 (26.3)	.53
	Program updates	235 (27.0)	<.001
	Service or resource promotion	89 (10.2)	.17
	Health education or promotion	93 (10.7)	.52
	Contest	32 (3.7)	.32
	Youth Achievement showcase	49 (5.6)	.001
	News link	92 (10.6)	<.001
	Social issue awareness raising	143 (16.4)	.79
	Campaign post	213 (24.4)	<.001
**Prevention topic**			
	Substance abuse	73 (8.4)	<.001
	Mental health	46 (5.3)	.10
	Safe sex	30 (3.4)	.02
	Sexually transmitted diseases	24 (2.8)	<.001
	Pregnancy prevention	31 (3.6)	.11
	Violence-bullying	29 (3.3)	.40
	Violence-fighting	13 (1.5)	.047
	Violence-partner	15 (1.7)	.20
**Positive youth development framework constructs**			
	Competence	173 (19.9)	.31
	Competence-physical activity	30 (3.4)	.005
	Competence-school	24 (2.8)	.50
	Competence-workplace	40 (4.6)	.003
	Confidence	172 (19.7)	<.001
	Connection	365 (41.9)	<.001
	Connection-family	38 (4.4)	.22
	Connection-peer	184 (21.1)	<.001
	Contribution	87 (10.0)	.87
	Contribution-action	33 (3.8)	.04

Adelante program participants comprised a mixture of very recently immigrated adolescents to the United States and slightly less recently immigrated youth. Given the makeup of the study community and program participants, Adelante staff emphasized the importance of using a mixed-language strategy for social media outreach, which mirrored in-person programming. Youth Facebook fans tended to show more engagement with posts that were either bilingual or in Spanish. Program update posts and campaign-specific posts incorporated the use of both Spanish and English, and this approach appears to have resonated well with this audience. Interestingly, there was no difference in engagement between posts that had a positive tone and those that had a negative tone.

**Table 3:** Results: post feature variables and user engagement.

Post feature (independent variable)		Posts, n (%)	*P* value (dependent variable)
**Multimedia content**			
	Video	88 (10.1)	.25
	Photo	574 (66.0)	<.001
	External link	193 (22.2)	<.001
**Language used**			
	English	241 (27.7)	.56
	Spanish	171 (19.6)	<.001
	Bilingual	380 (43.6)	.001
**Post tone**			
	Positive	480 (55.1)	.001
	Negative	82 (9.4)	.001
**People portrayed**			
	Female only	108 (12.4)	.05
	Male only	110 (12.6)	.43
	Male and female	254 (29.2)	<.001
	Group of youth	342 (39.3)	<.001
	Individuals	140 (16.1)	.61
	Adults	298 (34.2)	<.001

## Discussion

### Principal Findings

Social media use has become increasingly popular among young audiences, and Latino youth in the United States have been found to have nearly universal use of social media, making this method of communication essential for public health programs targeting this population [79,82]. Youth-oriented programs that incorporate social media outreach and engagement strategies extend beyond traditional in-person programming by opening a door to youth in the broader community who want and need health-related programming. This is particularly relevant for addressing health disparities among populations, such as recently arrived immigrants, who are more likely to be unaware of or disengaged from traditional health programming and potentially experience numerous barriers to participation.

Results for study aim 1 indicate that social media appears to be a useful tool for engaging Latino immigrant youth in health promotion programming. These levels of reach and engagement are on par with those seen in other studies examining health promotion efforts using social media [2,57-59,63-66]. Given that there are limited efforts targeting Latino immigrant adolescents through social media engagement, it is difficult to compare engagement observed in this study with that of other studies. However, this pilot effort serves as a starting point by which we can gauge reach and engagement of future efforts; it also provides guidance on content, features, and strategies to include in subsequent interventions. Despite the existence of numerous barriers to participation in traditional in-person programming, we were able to achieve a reasonable level of engagement through the Adelante Facebook page. This amplified the in-person programming, permitted the dissemination of branded prevention messages to youth, and combatted potential social, linguistic, and cultural isolation that this group of youth experiences through increased interaction with peers and program implementers in the Adelante digital network.

When characterizing social media consumption, Adelante youth Facebook fans tended to be more passive consumers of social media content, as opposed to active content contributors. Fans seemed more willing to interact with posts through clicks and likes but were potentially more hesitant to comment, share posts with their networks, or independently post user-generated content. This finding is consistent with other research and the phenomenon of “online identity management” described by Fergie et al, whereby youth described a complex vetting process to ensure that social media content they created was in line with their online persona [109]. Future youth programming should consider this finding and take steps to examine the concordance between youths’ personal brands and the established program brand, and how they intersect. Otherwise, this is a potential barrier for programs that ask youth to use their personal digital networks for dissemination of peer-to-peer prevention messages, such as the Living the Example youth ambassadors drug prevention program [110]. The more limited commenting and sharing by youth in our study may also indicate a potential hesitance among youth to be more actively engaged (sharing and commenting) on fan pages that are potentially viewable by a public audience. Future programs reaching high-risk youth populations should consider using Facebook closed groups instead of fan pages to increase the interactivity and engagement of youth in the group [111]. In contrast to our previous research with this population, which suggested that this audience would be interested in social media-based contests, contest-related posts did not produce significant user engagement. This may be explained by the audience’s patterns of passive social media consumption, discussed above. The contests sought to reward active engagement (likes or reactions, shares, comments, and user-generated content), but this call to action may have been incompatible with youth preferences of “lurking” on social media instead of engaging more actively. Future efforts should consider this potential tendency of passive social media consumption when determining programmatic targets and when deciding on mechanisms to increase engagement (eg, contests, posing questions, requesting post comments and shares, or use of closed groups).

Past research has identified some strategies for successful user engagement, including high sustained levels of social media activity [63] and targeted social media campaigns [64]. Results from study aim 2 highlighted that, for this audience, engagement was achieved both for posts that captured ongoing, day-to-day Adelante program activities and for planned, targeted social marketing campaign posts that featured local youth in campaign imagery. Building on this audience’s affinity for seeing themselves and their friends in posts, youth were more engaged in posts that highlighted program-related group activities that showed them and their friends having fun. These posts almost always included photos of program-related activities, which included interactive prevention workshops, academic tutoring, playing sports, user-generated video development, or being involved in other recreational activities. These posts also portrayed youth who were in the Facebook fans’ proximal peer networks and program activities taking place at familiar locations within their immediate community setting. The Adelante Facebook page served as a social extension of the in-person programming, where youth could see their friends and stay in touch. The success of certain post features, such as program updates or campaign posts, could be, in part, explained by the portrayal of recognizable youth from their community and school, and the use of local community visual imagery. For future efforts, we recommend collaboration with audience members in order to capture compelling imagery for posts and incorporation of audience-engaged content to result in higher engagement. We also recommend strategic incorporation of prevention messaging or other health promotion content into regular casual, habitual posts that keep participants connected with their friends. Furthermore, these findings bode well for interventions seeking to use peer-to-peer models for health promotion via social media.

Social media has also become an important mechanism for conveying health information, making audience engagement paramount for the utility of this strategy [5-7,31,47,60-62]. According to study aim 2 results, posts that resonated with this audience featured health and prevention information related to substance abuse, sexually transmitted disease prevention and safe sex, and violence prevention. Lack of engagement in certain topics, such as mental health and pregnancy prevention, was surprising, given that these topics were expressed as priorities by Adelante youth. Further analysis will be needed to explain diminished engagement for these topics. Regardless, we think that it is important for future efforts to carefully consider how health information can be packaged to increase the likelihood that young Latino immigrant audiences will consume this media and subsequently be exposed to the intended prevention messaging.

Our campaign formative research indicated that a mixed-language strategy would be the best option for this audience. For posts overall, Spanish-language and bilingual posts were more engaging than English-language posts, supporting our use of a mixed-language strategy, especially for communities that are diverse in terms of primary language spoken (English or Spanish) and have a large bilingual audience segment. Higher engagement with Spanish-language content for posts overall supports the finding from our formative research, where we were advised to “lean” toward more Spanish if we wanted to reach everyone, with the rationale that even English-dominant youth understand Spanish. Our prior research also suggested that the use of positive tone in posts would be more appealing to youth; however, we did not see any statistically significant differences in engagement between posts with a positive tone and posts with a negative tone.

### Limitations

The findings of this study should be interpreted in view of its limitations. Adelante intervention participants were predominantly Central American immigrants between the ages of 12 and 19 years living in a low-income, majority foreign-born, and Latino community. While study results are likely useful for research with other Latino adolescent subgroups, results may not necessarily be generalizable to these groups. Furthermore, while a sizeable proportion of Adelante Facebook fans were known participants in the program, some fans were not; it is possible that some fans who were engaged through social media outreach were not from our target audience in terms of geographic location, exact age group, or ethnicity.

Results should also be interpreted within the constraints of the study’s design; this social media outreach effort was a pilot demonstration, not a study of intervention outcomes. Thus, with no comparison group or behavioral measures, we are limited in the attribution of campaign effects and in the ability to determine health behavior change beyond initial engagement. Furthermore, youth from the target audience were users of numerous social media platforms, including Snapchat, Instagram, Twitter, and Kik Messenger, to name a few. The Adelante program did also cross-promote messages across platforms, particularly on Instagram and Twitter; however, this study examined only Facebook posts, which is likely an underrepresentation of audience engagement with overall Adelante social media outreach efforts.

### Conclusions

Results from this study indicated that the use of social media is a promising strategy for engaging young Latino immigrants in health promotion efforts. Through gaining an understanding of what post content and features are most appealing to young Latino audiences, social media outreach programmers can offer multiple opportunities for addressing health disparities among populations with additional barriers to engagement. Social media habits that include more passive consumption of posts should be considered when conceptualizing future social media outreach strategies and developing measures of engagement that are appropriate for these passive consumption habits. Interventions should consider approaches that can increase participant comfort with more active engagement, such as augmenting privacy through closed social media groups for certain activities where active engagement is sought. Additionally, social media prevention messages and posts should be created in collaboration with community youth, increasing the likelihood that the messages will resonate with this audience and will be compatible with youths’ social media engagement habits. This approach would also be more likely to result in posts with content and features that are most engaging for the intended audience.

The literature describes many efforts that seek to disseminate health-related messaging and engage audiences through formal social marketing campaigns. While our study confirmed the utility of campaign posts for youth engagement, our study also highlighted the high level of appeal of posts that covered more casual, day-to-day activities of program youth. Youth were particularly interested in social media posts insofar as they were an extension of the in-person programming: the youth could see the activities that were happening, see photos of themselves or their friends, and feel like part of this peer network. We recommend that programs include online efforts that intersect with in-person programming as a strategy to augment youth engagement. This is a relatively unexplored area that public health practitioners may consider as a mechanism for disseminating health promotion and risk prevention messaging.

## Abbreviations

PYD: positive youth development
